# Clinical impact of COVID-19 in a single-center cohort of a prospective study in cancer patients receiving immunotherapy

**DOI:** 10.2217/imt-2020-0211

**Published:** 2020-09-16

**Authors:** Melissa Bersanelli, Teresa Zielli, Fabiana Perrone, Chiara Casartelli, Fabiana Pratticò, Elena Rapacchi, Roberta Camisa, Michele Tognetto, Alberto Clemente, Diana Giannarelli, Sara Elena Rebuzzi, Alessandro Leonetti, Paola Bordi, Marcello Tiseo, Sebastiano Buti

**Affiliations:** ^1^Medical Oncology Unit, University of Parma, 43126, Parma, Italy; ^2^Medicine & Surgery Department, University of Parma, Parma, Italy; ^3^Biostatistics & Clinical Research Unit, Istituto Scientifico Romagnolo per lo Studio e la Cura dei Tumori (IRST) IRCCS, 47014, Meldola, Italy; ^4^Biostatistical Unit, Regina Elena National Cancer Institute, IRCCS, 00144, Rome, Italy

**Keywords:** anti-PD-1, cancer patients, COVID-19, immune checkpoint inhibitors, immunotherapy, influenza vaccine, interstitial pneumonia, SARS-CoV2

## Abstract

**Aim:** Evaluating the incidence and course of COVID-19 in cancer patients treated with immunotherapy. **Patients & methods:** We reported the influenza-like illness events with diagnosis of COVID-19 within the patient cohort enrolled in the prospective observational multicenter INVIDIa-2 study in the single center of Parma. **Results:** Among 53 patients, eight experienced influenza-like illness during the influenza season 2019/2020, and three of them had diagnosis of COVID-19. They were males, elderly, with cardiovascular disease. Radiological features of COVID-19 pneumonitis were found in all of three cases, although the pharyngeal swab resulted positive in only two. Two of these three patients died due to respiratory failure. **Conclusion:** Cancer patients are at high risk of severe events from severe acute respiratory syndrome coronavirus 2 (SARS-CoV-2) infection.

COVID-19, the disease caused by the new coronavirus SARS-CoV-2, was formally declared a pandemic on 1 March 2020 [1]. At the end of June, the disease had affected more than 10 million people, killing more than 500 thousand individuals worldwide [2]. Italy was the first European country to detect the disease in February 2020, then recorded an exponential growth of the contagion in the subsequent weeks. Emilia Romagna has been one of the most affected Italian regions, together with Lombardy and Veneto, reporting 25,426 cases and 3151 deaths overall on 30 April 2020 [3]. Meanwhile, the province of Parma alone (in Emilia Romagna) counted 3157 cases and 704 deaths due to COVID-19 [4].

Older age, comorbidities (e.g., diabetes mellitus, obesity, hypertension and heart disease) and history of cancer have been reported as factors associated with higher severity and lethality from SARS-CoV-2 infection [5,6]. Several retrospective studies suggest that cancer patients could be more vulnerable to SARS-CoV-2 infection compared with the general population, due to the immunosuppressive state generated by systemic cancer treatments and the malignancy itself [7–11].

In the last few years, the cancer treatment landscape has undergone a profound change. Immune checkpoint inhibitors (ICIs) are currently the standard of care in several types of advanced or metastatic solid tumors [12]. While the immunosuppressive effect of chemotherapy is established, the debate is still open about the real impact of infections in patients treated with ICIs [13]. ICIs can restore the cellular immunocompetence, but they could also trigger the cytokine-release syndrome, a rare phenomenon of immune hyperactivation that could theoretically exacerbate a cytokine storm during COVID-19 [14,15].

Furthermore, the concurrency of seasonal influenza and COVID-19 epidemics is becoming a major concern for public health and even more for cancer patients [16].

The INVIDIa-2 study, a prospective, multicenter, observational trial promoted in Italy by the Federation of Italian Cooperative Oncology Groups, was coordinated by the Medical Oncology Unit of Parma. It was designed to investigate the clinical efficacy of influenza vaccination in advanced cancer patients receiving ICIs from October 2019 to January 2020. The study, whose primary end point was the influenza-like illness (ILI), provided the differential diagnosis of all ILI events, detecting all the symptomatic COVID-19 cases in the enrolled population.

Herein, we reported a preliminary single-centre analysis, considering only the patient population enrolled in the study in the center of Parma, focusing on the clinical characteristics and the course of SARS-Cov-2 infections in patients developing ILI diagnosed with COVID-19.

## Material & methods

The INVIDIa-2 study is a multicenter, prospective, observational study, still ongoing in the follow-up phase. All patients with advanced solid tumors treated with ICIs (alone or in combinations) at the time of enrollment, from 1 October 2019 to 31 January 2020, namely during the influenza vaccinal season, were eligible. Patients were enrolled regardless of the decision to have or not the influenza vaccine, left to clinical practice, namely to the individual patient decision and to the general practitioner advice.

The influenza season, from October 2019 to April 2020 included, constituted the ILI observation period for the enrolled patients, with a cut-off date of 30 April 2020 for the primary end point and the secondary end points regarding the severity and fatality of ILI, immune-related toxicity and adverse reactions to the vaccine.

The secondary end points regarding treatment outcomes and overall survival are being investigated with a longer follow-up.

The study involved 82 Italian centers, with an enrollment target of 974 patients according to the sample size calculation; an over-accrual of 30% was provided basing on the expected rate of screening failures.

Inclusion criteria were: age >18 years; signing of the informed consent form for the study; subject is cooperative and compliant vis-à-vis ILI monitoring and is willing to provide the date of any influenza vaccination (which the patient may have at his/her discretion) and the type of vaccine used; diagnosis of advanced or metastatic solid tumor; indication to start systemic therapy with ICIs (PD-1 inhibitors, PD-L-1 inhibitors, or CTLA-4 inhibitors) as a single agent or in combination with other ICIs or with other cancer treatments (including chemotherapy agents, tyrosine kinase inhibitors, other monoclonal antibodies or compounds with different mechanisms of action) by 31 January 2020, as part of routine clinical practice or clinical studies with an immunotherapy agent or systemic immunotherapy already in progress at the time of enrollment provided it was started no earlier than 1 April 2019.

The exclusion criteria for the study were: blood cancers or lymphomas; immunotherapy treatment line commenced prior to 1 April 2019; treatment as part of a blinded clinical trial in which an immunotherapy arm is compared with an arm not receiving immunotherapy (patients treated in blinded studies are eligible provided immunotherapy is administered in all treatment arms, either alone or in combination with other agents).

ILI was defined, according to the European Centre for Disease Prevention and Control definition [17], as a sudden and rapid onset of one or more of the following general symptoms: fever or low-grade fever, malaise and/or listlessness, headache, myalgia; and at least one of the following respiratory symptoms: cough, sore throat, dyspnea. ‘Sudden and rapid’ is taken to mean to the extent that the date of symptom onset can be clearly identified. Confirmed influenza was defined as confirmed by pharyngeal/nasal swab or serology tests.

All the ILI episodes, laboratory tests, complications, hospitalizations and pneumonitis were recorded. Therefore, the study prospectively recorded all the COVID-19 ILI events within the enrolled patient population.

Patients were included in this non-prespecified COVID-19 preliminary analysis, if potentially exposed to Sars-Cov-2 infection, namely alive on 31 January 2020, when the Italian government declared the National emergency. The incidence of COVID-19 was assessed among patients with ILI symptoms. Confirmed COVID-19 cases were defined with Sars-CoV-2 nasal and/or pharyngeal swab positivity according to the local clinical practice. COVID-like cases were defined as clinical and radiological characteristics with high suspicion for COVID-19, irrespective of the laboratory confirmation, considering both the epidemiological context (local outbreak peak) and the pathognomonic clinical and radiological features.

The study was approved by the Local Ethical Committee and was conducted in accordance with the precepts of Good Clinical Practice and the declaration of Helsinki.

## Results

### Overall population characteristics

Overall, 59 patients were enrolled in the study in the coordinating center of Parma. Of them, 53 patients matched the eligibility criteria for the COVID-19 analysis and were included in the present report.

Patients characteristics are summarized in Table 1. Median age was 69 years (34–90); 25 out of 53 were elderly patients (age >70 years). The majority were men (77%) and current or former smokers (81%). Primary tumors were: lung cancer (54.7%), melanoma (16.9%), renal cell carcinoma (13.2%), urothelial carcinoma (7.5%), others (7.5%). Most of the patients had at least one comorbidity. Hypertension was the most common (52.8%), followed by chronic obstructive pulmonary disease (39.6%).

**Table 1.** Baseline characteristics of the overall case series.

	Patients, n = 53 (100%)
**Age, n (%)**	
Median	69 (34–90)
Elderly (>70 years)	25 (47.2%)
**Sex, n (%)**	
Male	41 (77%)
Female	12 (23%)
**ECOG PS, n (%)**	
0	40 (75.5%)
1	12 (22.6%)
2	1 (1.8%)
**Comorbidity, n (%)**^†^	
Hypertension	28 (52.8%)
Other cardiovascular disease	6 (11.3%)
Chronic obstructive pulmonary disease	21 (39.6%)
**Smoking status, n (%)**	
Smoker	17 (32%)
Former	26 (49%)
Never	10 (18.8%)
**Primary tumors, n (%)**	
Lung	29 (54.7%)
Melanoma	9 (16.9%)
Kidney	7 (13.2%)
Urothelial	4 (7.5%)
Others	4 (7.5%)
Immunotherapy line, n (%)	
First-line	38 (71.7%)
Second-line or more	15 (28.3%)
**Immune checkpoint inhibitor administered, n (%)**	
Pembrolizumab	20 (37.7%)
Nivolumab	9 (17%)
Atezolizumab	9 (17%)
Chemotherapy + pembrolizumab	4 (7.5%)
Others	11 (20.7%)

† 11 patients presented more than one comorbidity.

ECOG PS: Eastern Cooperative Oncology Group Performance status.

In this single-center population, 37 patients (69.8%) received the flu vaccination during the vaccination season 2019/2020.

**Figure 1.**
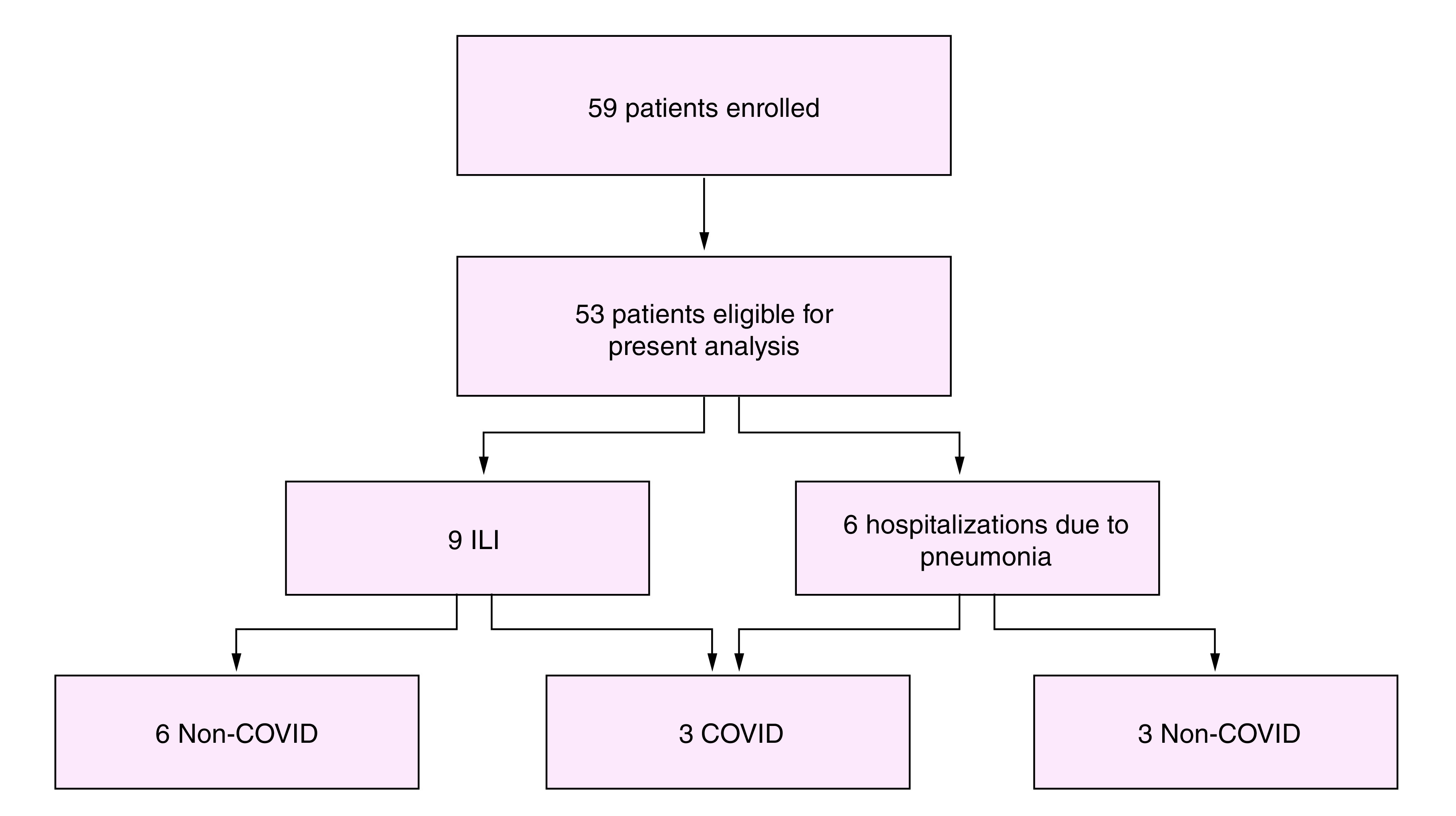
Flow diagram of the cases included in the present analysis according to the end points of interest. ILI: Influenza-like illness.

Among 53 patients enrolled, eight experienced ILI in the prospective follow-up time frame, during the influenza season 2019/2020. For five patients, the ILI event occurred between 1 November 2019 and 31 January 2020, that was before the SARS-CoV-2 outbreak. Obviously, these patients did not undergo SARS-CoV2 laboratory testing. Nevertheless, their A/H3N2 flu swab was negative. The other three patients, named herein as patient 1, 2 and 3, developed ILI during the COVID-19 pandemic (after 31 January 2020). Two of them were defined as ‘confirmed COVID-19’ due to swab positivity, while the third was defined as ‘COVID-like ILI’ due to clinical and radiological diagnosis of COVID-19 interstitial pneumonia, with typical clinical features, despite the negative result of the real-time PCR (RT-PCR) of the nasal/pharyngeal swab (Figure 1).

Of six patients with clinical and radiological diagnosis of pneumonitis requiring hospitalization, three patients presented radiological features consistent with COVID-19 pneumonia.

The occurrence of COVID-19 was less frequent among patients vaccinated against influenza (3.7 vs 11% in unvaccinated patients).

At the data cut-off, on 30th April, six deaths occurred, of which four were due to disease progression and two to COVID-19.

Results regarding Influenza A- and B-related ILI are not yet available because they are the objective of an ancillary study (INVIDIa-bios study), which is currently still ongoing.

### Characteristics of COVID-related ILI patients

Patients 1 and 2, who experienced confirmed COVID-related ILI and patient 3, who experienced COVID-like ILI, were affected, respectively, 1 by locally advanced and 2 by metastatic non-small-cell lung cancer. They were all males, the age was 73 years in patients 1 and 2, 69 years in patient 3. Patients 1 and 2 had a history of hypertension. In addition, patient 1 was affected by chronic obstructive pulmonary disease, atrial fibrillation and cerebrovascular disease and patient 2 by concomitant pleural mesothelioma. Patient 3 did present only chronic obstructive pulmonary disease. Patients 2 and 3 were active smokers at the time of the infection, patient 1 discontinued smoking more than 1 year before.

The ICI received was pembrolizumab in two of the cases, after progression to platinum-doublet chemotherapy in patient 2 and as first-line treatment in patient 3. Patient 1, who was treated with durvalumab after exclusive chemo-radiation treatment for locally advanced disease, discontinued ICI because of immune-related adverse events in October 2019. None of the patients received further therapy after ICI before developing COVID-19.

Patients 2 and 3 did not receive the flu vaccine. On the other hand, patient 1 received influenza vaccination as well as pneumonia, diphtheria, tetanus, pertussis and meningococcus vaccines, given his clinical history of splenectomy.

The time between the last cycle of immunotherapy received by the patient and the onset of ILI symptoms was 135 days, 6 days and 56 days in patient 1, 2 and 3, respectively. All three patients complained the occurrence of fever and dyspnea, requiring admission to the Emergency Department. In addition, dry cough and diffuse myalgias were experienced by two patients.

High-resolution computed tomography (HRCT) allowed the diagnosis of interstitial pneumonia in all three patients. The visual score, representing the percentage of the lung volume affected by pneumonitis, was of 30% in patient 1, 50% in patient 2 and 40% in patient 3 [18].

**Figure 2.**
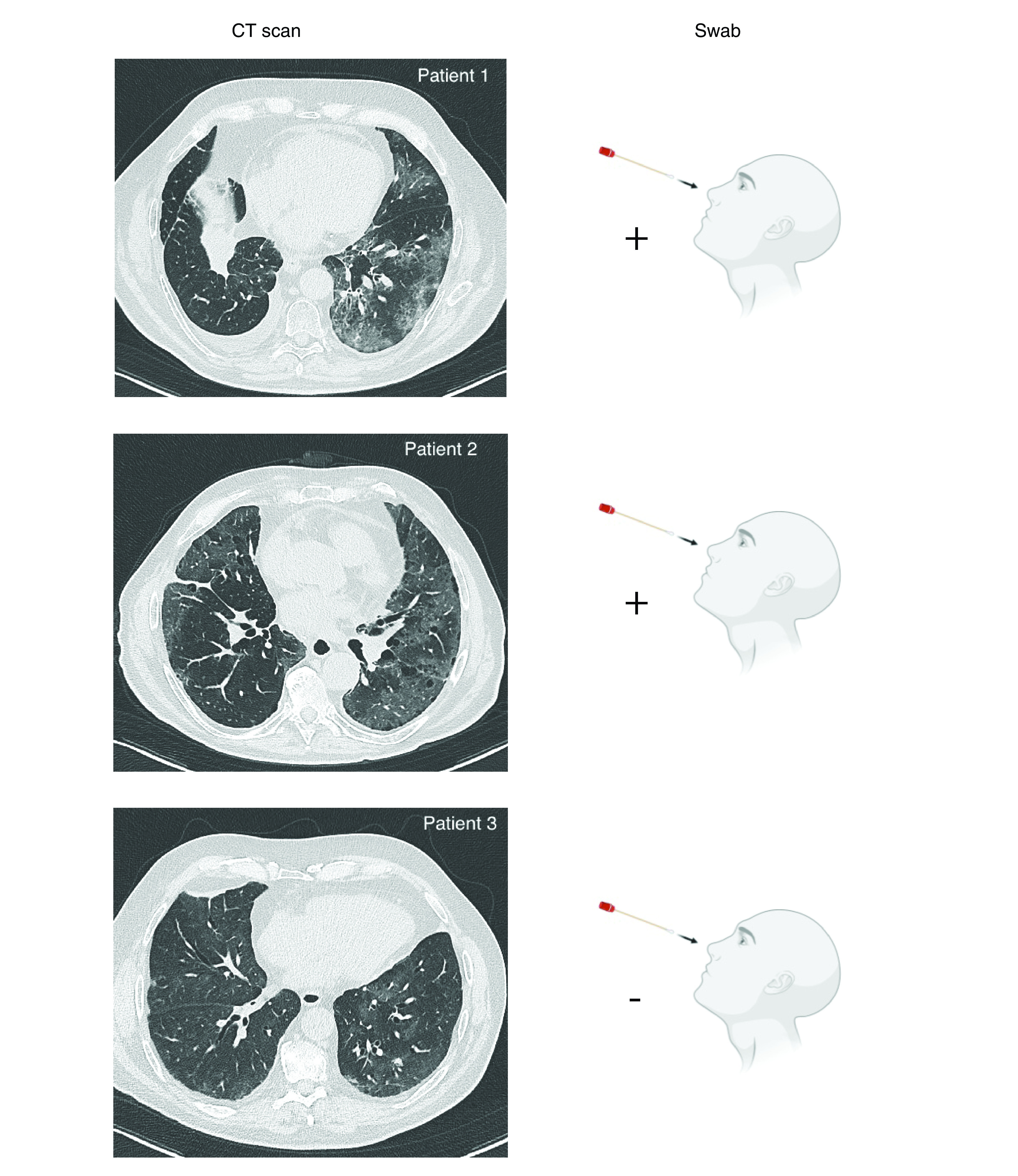
CT scan imaging of patients 1, 2 and 3. Ground glass opacities, with peripheral and subpleural distribution, and the involvement of multiple lobes, mainly the lower lobes, are suggestive for COVID-19-related pneumonia. SARS-CoV-2 nasal-pharyngeal swab was positive in patients 1 and 2 and negative in patient 3.

**Figure 3.**
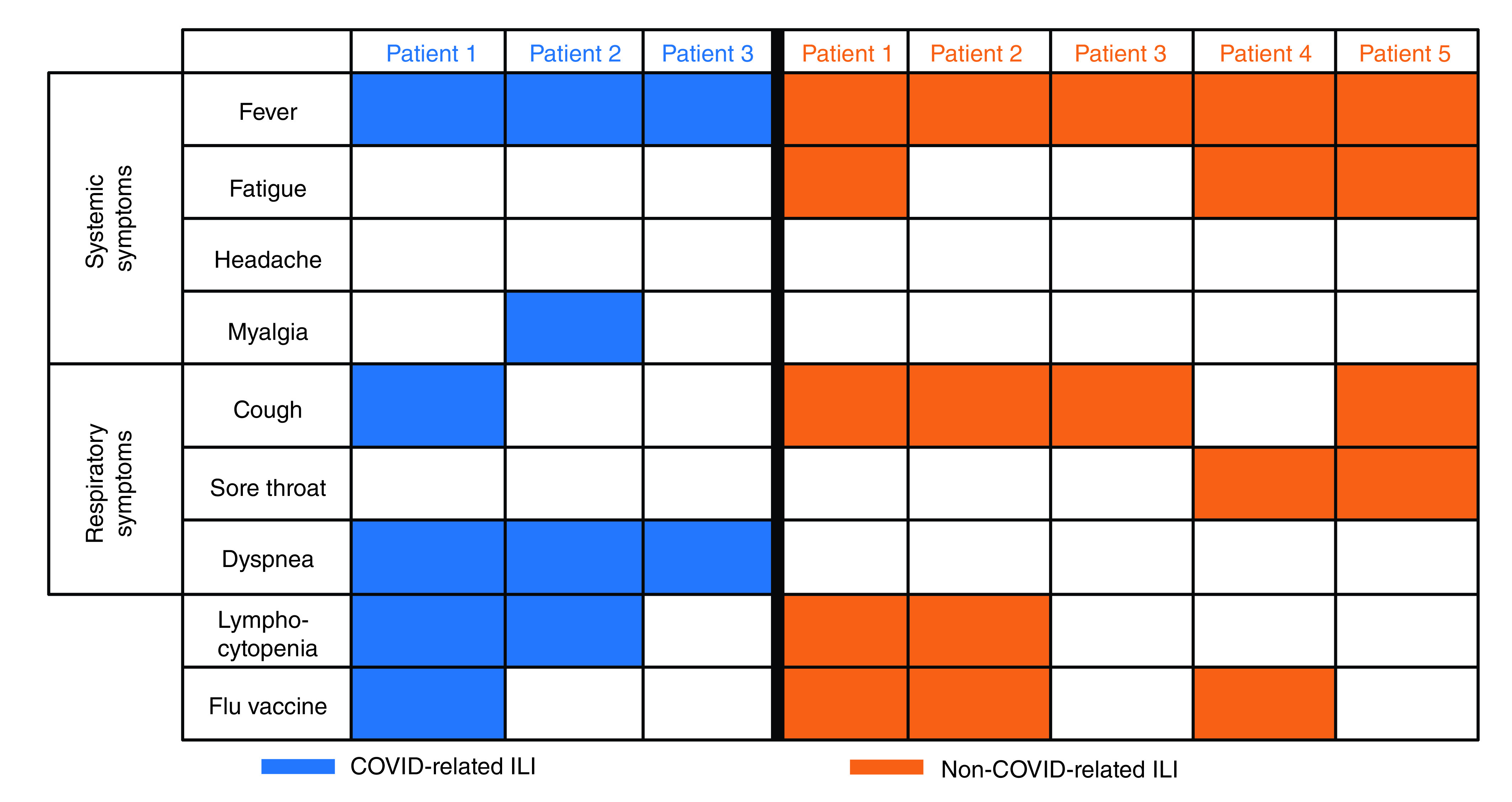
Clinical characteristics of COVID-related and not-COVID related influenza-like illness. ILI: Influenza-like illness.

The radiological findings were the same in all three patients: ground glass opacities, with peripheral and subpleural distribution, and the involvement of multiple lobes, mainly the lower lobes (Figure 2). A nasopharyngeal swab with RT-PCR diagnostic assay was performed in all cases and resulted positive for SARS-CoV-2 in patients 1 and 2. Despite negative swab, radiological features and clinical presentation, together with the epidemiologic contextualization, led to the clinical diagnosis of COVID-19 in patient 3. In the blood samples, the new onset of absolute lymphocytopenia was observed in patients 1 and 2, at the time of the infection. The characteristics of the three patients are summarized in Figure 3.

All patients required hospitalization due to severe respiratory failure and patients 1 and 2 died after 6 days and 18 days, respectively, from the diagnosis of pneumonitis, due to acute respiratory distress syndrome. Patient 3 was alive on 30 April 2020.

## Discussion

To our knowledge, the INVIDIa-2 trial is the first study prospectively detecting symptomatic COVID-19 in advanced cancer patients treated with ICI.

In our single-center population, the COVID-19 incidence was 5.7% and the COVID-related mortality rate was 3.7%. The incidence of the disease in our case series was high, especially considering that, on 30th April, the overall incidence rate in the general population of the Parma province was 0.7% [19]. On the other hand, we should consider that the original study was designed to detect the ILI. Only patients experiencing influenza-like symptoms were included in the ILI differential diagnosis, and thus in the COVID-19 detection. Asymptomatic cases, even with positive SARS-CoV-2 swab, were omitted, and only COVID-19 symptomatic cases were collected. Moreover, our case series included patients with clinical characteristics known to be associated to high risk of severe events from COVID-19, as locally advanced and metastatic cancer and frequent access to the hospital [7].

The treatment received by our case series was based on ICIs. The evidence about if and how immunotherapy could contribute to the risk of contagion form SARS-CoV-2 and of higher severity of COVID-19 is controversial. Among retrospective studies published, Dai *et al.* suggested that Chinese cancer patients treated with ICIs had a higher death rate and a higher chance of developing critical illness compared with cancer patients receiving other treatments (chemotherapy and radiotherapy). Nevertheless, the sample size of the study subgroups was very limited, as only six patients received immunotherapy [7].

Otherwise, the results of the TERAVOLT study in lung cancer patients with COVID-19, showed that the administration of immunotherapy was not associated with increased risk of death. On the contrary, receiving chemotherapy alone was a risk factor for death from COVID-19 [10].

Recently, Szabados and colleagues published a case series, exploring the incidence of COVID-19 among 74 patients affected by genitourinary cancers on ICIs treatment. In this report, 11 patients experienced symptoms related to COVID-19 and in four of these cases the swab was positive for SARS-CoV-2 infection. The authors concluded that the higher risk of COVID-19 death associated with systemic therapy in cancer may not apply to patients on ICIs [20]. On the other hand, we interpreted such data as a potential lower lethality of the disease in genitourinary malignancies, when compared with lung cancer, which is to be considered a risk factor in itself [21].

All the reports mentioned above were retrospective and the patient populations were heterogeneous. Therefore, these findings are not sufficient to solve the debate.

All the three patients with COVID-19 in our series had clinical characteristics associated to high risk of morbidity from COVID-19, such as older age, male sex and cardiovascular disease. Moreover, all patients were affected by lung cancer. Several studies demonstrated that lung cancer patients are more likely to develop severe anoxia and to progress more rapidly to severe forms of COVID-19, given their worse baseline pulmonary function [22].

A spectrum of symptoms has been reported in SARS-CoV-2-infected patients. The main manifestations are fatigue, fever, dry cough, myalgia and dyspnea, with less common symptoms being nasal congestion, headache, runny nose, sore throat, vomiting and diarrhea [23]. Anosmia and dysgeusia have been also described [24]. Although the symptoms are similar both in the general population and in cancer patients, dyspnea occurs earlier in patients affected by tumors [19]. In our results, dyspnea was the only symptom complained by all of the patients affected by COVID-related ILI compared with not COVID-related ILI.

HRCT scan and laboratory tests allowed the diagnosis of COVID-19 disease in our cases. According to the local clinical practice, the only test used to detect active infection from SARS-CoV2 was RT-PCR to identify the viral RNA on nasal and/or pharyngeal swabs [25,26]. These tests are highly specific and moderately sensitive, with a respective sensitivity of 63 and 32% for pharyngeal and nasal swabs [27]. The HRCT scan allowed the diagnosis of COVID-19 pneumonia regardless of the swab results. The typical radiological findings, in our study as well as in the previous literature, included ground-glass opacities, patchy consolidations, interlobular septal thickening, reticular appearance and fibrous strips [28]. Patchy consolidations are a risk factor for developing severe respiratory problems [29]. Peripheral ground glass opacities were found either in patients with positive and negative swab. Thus, if a positive swab is diagnostic for SARS-CoV-2 infection, a negative one does not rule out the disease and a CT scan should always be performed in symptomatic patients. Sputum and bronchoalveolar lavage are alternative tools for the SARS-CoV-2 detection. These invasive tests showed high positivity rates in at-risk population (93 and 72%, respectively), but their application in clinical practice is limited by feasibility issues [27].

Many non-specific inflammatory markers, including C-reactive protein (CRP), erythrocyte sedimentation rate and ferritin, have been reported as elevated in SARS-CoV-2-infected patients [30]. Factors associated with poor outcomes from COVID-19 are lymphopenia, high levels of D-dimer, cardiac troponin I, serum ferritin, lactate dehydrogenase and IL-6 [22,31]. In particular, the reduced lymphocyte count could be subtended by the cytokine storm. This syndrome is characterized by increased level of pro-inflammatory interleukins (mostly IL-6, IL-2, IL-7, GCSF, IFN-γ inducible protein 10, MCP-1, MIP1-a) and TNF-α, which may promote lymphocyte apoptosis [32]. Considering this background, immunosuppressive attempts have been investigated against COVID-19, like previously happened in the case of other coronaviruses such as SARS-CoV and Middle East respiratory syndrome coronavirus (MERS-CoV) [33].

The limitations of our study are represented by the small sample size and the small subgroups, not allowing inferential statistics. Therefore, despite the reliability of a prospective case series, no definitive conclusions can be drawn about impact of flu vaccination on COVID-19 infection. We could only hypothesize that the flu vaccine could minimize the severity of COVID-19 disease.

## Conclusion

Although several retrospective studies have been published since the outbreak of SARS-CoV-2, the knowledge about COVID-19 impact in cancer patients is still limited.

Our results suggest that cancer patients are at high-risk of getting the infection and of developing severe events from COVID-19. The lack of a control group receiving chemotherapy, radiotherapy or targeted therapy do not allow any conclusions about the role of the systemic treatment in the morbidity of the disease.

Further prospective studies would be deserved in the case of future new COVID-19 outbreaks, to define the true impact of the infection in cancer patients according to their systemic treatment, in order to help the clinical oncologist in the management of the cancer therapy during the pandemic.

**Summary points**

- Cancer patients are a very vulnerable population to symptomatic severe acute respiratory syndrome coronavirus 2 (SARS-CoV-2) infection.
- A positive swab allows the diagnosis of COVID-19, but a negative swab does not exclude the infection. CT scan should also be performed.
- No definitive conclusions are possible about the impact of flu vaccination on the occurrence and severity of COVID-19.
- No recommendations can be currently provided about the continuation of treatment with immune checkpoint inhibitors during COVID-19 outbreaks.
